# Randomized single-dose crossover comparative bioavailability study of two novel oral cannabidiol (CBD) formulations in healthy volunteers under fed conditions, compared to a standard CBD isolate capsule

**DOI:** 10.1186/s42238-025-00312-9

**Published:** 2025-08-06

**Authors:** Mehmet Nevzat Pisak, Edibe Bereket, Aydin Erenmemisoglu

**Affiliations:** 1 Imuneks Farma Pharmaceuticals Industry and Trade Inc, Istanbul, Türkiye; 2 Alpan Farma Ar Ge Biyot.Ltd, Kayseri, Türkiye

## Abstract

**Background:**

This study aimed to compare the pharmacokinetics and relative bioavailability of two novel cannabidiol (CBD) formulations including bioavailability-enhanced capsule (CBDNEXT Supra capsule) and a bioavailability-enhanced liquid (CBDNEXT Supra liquid)– against a standard high-purity CBD isolate capsule in healthy volunteers under fed (low-fat) conditions.

**Methods:**

A single-dose, open-label, randomized, three-period, three-sequence crossover trial was conducted in 12 healthy male volunteers (18–50 years). Of the 12 enrolled subjects, 9 completed all three treatment periods per protocol. Each received 40 mg of CBD as one of three formulations: Test-1 (enhanced CBD capsule), Test-2 (enhanced CBD liquid), or Reference (unformulated CBD isolate capsule with microcrystalline cellulose only as excipient), with a washout of 14 days between treatments. All doses were administered 30 min after a standardized low-fat (~ 300–350 kcal, < 10 g fat) breakfast to minimize the impact of dietary fat on CBD absorption. Blood samples were collected up to 72 h post-dose for plasma CBD and 7-hydroxy-CBD analysis by a validated LC–MS/MS method (LLOQ 0.1 ng/mL). Pharmacokinetic (PK) parameters (C_max, tmax, AUC_0–72, and t_1/2) were determined by non-compartmental analysis. Safety was assessed by adverse events (AEs), vital signs, and laboratory tests (including liver enzymes). Plasma cortisol was measured pre-dose and at 2-, 4-, and 8-hours post-dose as an exploratory pharmacodynamic marker.

**Results:**

Nine subjects completed all treatments (three withdrew: two for positive drug screens and one for an intercurrent moderate infection). Mean plasma CBD concentration–time profiles for the three formulations demonstrated markedly different absorption kinetics. The enhanced capsule achieved the highest peak CBD concentration (C_max 14.1 ng/mL) and exposure (AUC_0–72 38.0 h·ng/mL), compared to the enhanced liquid (C_max 6.2 ng/mL, AUC_0–72 20.2 h·ng/mL) and the reference capsule (C_max 2.4 ng/mL, AUC_0–72 11.7 h·ng/mL). The time to peak concentration (tmax) was shortest for the liquid (median ~ 1.0 h), followed by the capsule (2.0 h), and longest for the reference (6.0 h). Inter-individual variability in C_max and AUC was substantially lower for both novel formulations (coefficients of variation ~ 27–44%) than for the reference (> 90%). Statistical analysis confirmed that both the enhanced capsule and liquid produced significantly greater C_max and AUC_0–72 than the reference (geometric mean C_max ratios ~ 566% and ~ 248%, AUC_0–72 ratios ~ 328% and ~ 166%, respectively; 90% confidence intervals did not include 100%). The 7-hydroxy-CBD metabolite reached much lower plasma levels than parent CBD for all treatments (C_max 0.4–1.8 ng/mL, ~ 5–13% of parent C_max), with a slightly delayed tmax (~ 2–6 h) and similar elimination half-life. No serious AEs occurred. The only treatment-related AE was mild-to-moderate transient headache in four subjects (1 on capsule, 2 on liquid, 1 on reference), all resolved without intervention. No clinically significant changes in liver function tests or other laboratory values were observed. Plasma cortisol levels remained within normal ranges after all treatments, with a decline over 8 h consistent with the normal diurnal rhythm and no significant differences between formulations.

**Conclusions:**

Both novel formulations markedly improved CBD oral bioavailability under low-fat fed conditions relative to an unformulated CBD isolate capsule. The enhanced capsule in particular achieved a ~ 5.7-fold higher C_max and ~ 3.3-fold higher AUC than the reference, while the enhanced liquid was absorbed ~ 2.5-fold faster. Variability was reduced with the new formulations, and both were well tolerated at the 40 mg dose. These results indicate that the surfactant/acid formulation technology effectively enhances CBD absorption even with a low-fat meal, potentially obviating the need for high-fat co-administration. Further clinical studies (including in female subjects and at steady state) are warranted to confirm these pharmacokinetic advantages in broader populations.

**Trial registration:**

Not prospectively registered (pilot pharmacokinetic study without therapeutic intent, thus not subject to mandatory registration).

**Supplementary Information:**

The online version contains supplementary material available at 10.1186/s42238-025-00312-9.

## Introduction

*Cannabis sativa* L. produces numerous phytochemicals, most notably Δ^9^-tetrahydrocannabinol (THC) and cannabidiol (CBD), along with various terpenes and flavonoids. Unlike psychoactive THC, CBD is non-psychotropic and has attracted growing interest for its potential therapeutic uses in conditions such as epilepsy, anxiety, pain, and inflammation. (Pertwee 2008) However, CBD’s oral bioavailability is notoriously poor, typically estimated at < 10–20% in humans (Millar et al. 2018). This is largely due to its extremely low water solubility and extensive first-pass metabolism, causing orally administered CBD to be prone to pre-systemic elimination and resulting in highly variable absorption with often sub-therapeutic plasma levels (Perucca and Bialer 2020). Overcoming these formulation challenges is critical to maximize CBD’s clinical benefits.

Various strategies have been explored to improve CBD’s oral bioavailability, including co-administration with high-fat meals and the use of lipid-based delivery systems, cyclodextrin inclusion complexes, and nano-emulsions (Hegde et al. 2022). For instance, taking CBD with a high-fat meal can increase systemic exposure by around four-fold compared to fasting (Birnbaum et al. 2019). However, reliance on dietary fat is impractical for consistent dosing and introduces variability (Zgair et al. 2016). More advanced formulations have been developed to enhance CBD solubility independent of food. A gelatin matrix pellet formulation (PTL101, 10 mg) achieved a C_max of ~ 3.2 ng/mL and AUC ~ 9.6 h·ng/mL, and a simple medium-chain triglyceride (MCT) oil solution of CBD (25 mg) yielded only ~ 3.1 ng/mL C_max and ~ 9.5 h·ng/mL AUC. These examples illustrate the substantial improvements possible with formulation technology (Atsmon et al. 2018).

In this context, Imuneks Farma (Turkey) has developed a novel “CBDNEXT Supra” formulation technology in both solid capsule and liquid forms. The technology is based on a patented composition comprising CBD isolate combined with pharmaceutically acceptable excipients designed to create a micro-emulsion upon ingestion, thereby vastly increasing CBD’s apparent solubility. In particular, the formulations include a high hydrophilic–lipophilic balance (HLB) surfactant (emulsifier), an organic acid, and a polysaccharide/dextrin component. For example, one embodiment mixes CBD with polysorbate 80 (a nonionic surfactant), stearic acid, and maltodextrin. The surfactant helps form stable nano-droplets of CBD in aqueous environments; the acid may promote ionization or improved dissolution of CBD; and the dextrin and colloidal silica) can facilitate a solid dispersion and prevent aggregation. Together these components address the solubility and permeability limitations of oral CBD, as demonstrated in preclinical development with significantly enhanced dissolution rates and absorption (as detailed by the patent application EP3886839A1) (Pisak 2021).

Mounting clinical evidence indicates that systemic CBD exposure is directly associated with therapeutic benefit. In pivotal trials of the EMA-approved oral solution Epidyolex^®^ (100 mg mL⁻¹ ethanol/sesame-oil vehicle), higher dosage regimens (20 mg kg⁻¹ day⁻¹) achieved greater seizure-frequency reductions than 10 mg kg⁻¹ day⁻¹, and responder rates correlated with trough-plasma CBD concentrations (Devinsky et al. 2017; Abu-Sawwa et al. 2020). Yet Epidyolex requires large millilitre volumes (up to 1.4 g CBD for a 70 kg patient) because its absolute oral bioavailability remains low and highly variable (intra-subject CV ≈ 45–67%) even when administered with food (Taylor et al. 2018). This dose burden contributes to gastrointestinal adverse events, complicates paediatric administration, and inflates treatment cost. Consequently, formulations that raise CBD bioavailability and lower variability could deliver the same—or superior—efficacy at markedly lower milligram doses, improving tolerability and adherence while reducing cost. The present study therefore investigates whether the CBDNEXT Supra technologies can bridge this translational gap by delivering higher, more reproducible plasma exposures compared with the current EMA-approved benchmark as a pilot exploratory study.

The present study aimed to evaluate pharmacokinetics and relative bioavailability of two such novel CBD formulations– a 40 mg CBD enhanced-release powder capsule and a 40 mg/10 mL CBD enhanced-release liquid vial (CBDNEXT Supra products)– relative to an unformulated 40 mg CBD isolate capsule used as a reference. We conducted a single-dose crossover trial in healthy volunteers under fed, low-fat conditions. A low-fat meal was chosen to minimize the confounding effect of high dietary fat on CBD absorption, allowing a clearer assessment of the formulation effects. With the reference capsule only containing CBD combined with microcrystalline cellulose. Notably, most published studies reporting high oral CBD exposure have involved either much larger doses or high-fat co-administration. By using a moderate 40 mg dose and a light breakfast, we aimed to simulate real-world usage (e.g., dosing with a regular meal) while challenging the formulations to enhance bioavailability without the “boost” of heavy fats or ethanol (as in Epydiolex). We also measured plasma cortisol as an exploratory pharmacodynamic outcome, given literature suggesting CBD might modulate acute stress responses and cortisol levels. The study was designed and is reported in accordance with CONSORT 2010 guidelines for randomized trials and the European Medicines Agency (EMA) Guideline on the Investigation of Bioequivalence. (Schulz et al. 2010; EMA 2010)

## Materials and methods

### Study design and ethics

This was a single-center, open-label, randomized, three-treatment, three-period, three-sequence crossover study in healthy adult volunteers. Each participant received a single oral dose of each of the three CBD formulations (two test formulations and one reference) in a randomized sequence, with washout intervals of 14 days between periods. The study protocol was approved by the Institutional Review Board/Independent Ethics Committee/(lRB/IEC) at APIC/PRU clinical site. The trial was conducted in accordance with International Council for Harmonisation of Technical Requirements for Pharmaceuticals for Human Use (ICH) guidelines. While the study adhered to ethical principles, it was not prospectively registered in a publicly accessible database before recruitment of the first subject. In addition, the trial adhered to the EMA Guideline on the Investigation of Bioequivalence (CPMP/EWP/1401/98 Rev.1/Corr) for crossover bioavailability studies. All participants provided written informed consent before enrolment. No protocol amendments were made after study initiation, and there was no patient or public involvement in the design, conduct, or reporting of this study.

### Subjects

Healthy male volunteers aged 18–50 years with body mass index 18–30 kg/m^2^ were eligible for inclusion. Key inclusion criteria included the absence of any significant medical conditions on history or physical examination, normal baseline laboratory test results, and no use of any cannabinoids or other drugs prior to the study. Key exclusion criteria were positive serology for HBsAg, anti-HCV, or anti-HIV-1/2 antibodies and any history of alcohol or substance abuse, positive tests for drugs of abuse, smoking, or use of medications/supplements that could affect CBD metabolism. To ensure compliance, subjects underwent urine screens for benzodiazepines, cocaine, cannabis, amphetamines, and opioids, as well as breath alcohol tests at admission for each study period. Twelve subjects were enrolled to meet the EMA recommendation of ≥ 12 evaluable subjects in crossover bioequivalence work; although three withdrew, the observed intra-subject CV of log-AUC (18%) still yielded > 80% post-hoc power, satisfying the intent of the guideline. Subjects were randomized to one of six possible treatment sequences (ABC, ACB, BAC, BCA, CAB, CBA, where A = Test-1, B = Test-2, C = Reference) using a computer-generated randomization list. Due to the nature of the formulations (capsule vs. liquid), blinding was not feasible (open-label design).

Of the 12 enrolled subjects, 9 completed all three treatment periods per protocol. Three subjects did not complete the study: two were withdrawn after Period 1 due to unexpected positive urine cannabinoid tests at the start of Period 2 (indicating undisclosed cannabis use between periods), and one subject was withdrawn before Period 3 due to a moderate adverse event (a transient viral infection with fever, deemed unrelated to the study drug). The pharmacokinetic analysis population thus consisted of 9 completers (all males; age range 19–44 years). All 12 subjects were included in the safety evaluation for the treatments they received (11 subjects contributed safety data for at least one dose). No protocol deviations occurred aside from the noted withdrawals. Although only nine subjects completed, the observed intra-subject CV for log AUC_0–72 was ~ 18%, yielding > 80% post-hoc power; thus, nine completers satisfied the intent of the EMA guideline in terms of adequate power.

### Formulations and dosing

#### Test product 1 (Enhanced CBD Capsule)

A 40 mg CBD oral hard gelatin capsule formulated with the CBDNEXT Supra technology (Imuneks Farma, Turkey). Each capsule contained 40 mg of purified CBD isolate (99.8% purity, Medropharm AG, Switzerland) dispersed in a proprietary mixture of excipients designed to promote micellar solubilization of CBD in the gastrointestinal tract. The formulation included a high-HLB nonionic emulsifier (surfactant), an organic acid, and a polysaccharide carrier, based on a patented composition. In practice, the proprietary composition consists of CBD adsorbed onto a solid matrix with polyoxyethylene sorbitan fatty acid esters (e.g., polysorbate 80), maltodextrin, stearic acid, and a silica derivative. The capsule is intended to disintegrate rapidly in the stomach, releasing CBD in a solubilized form.

#### Test product 2 (Enhanced CBD Liquid)

A 40 mg/10 mL CBD oral liquid (CBDNEXT Supra Liquid, Imuneks Farma, Turkey). This formulation contained 40 mg of CBD isolate dissolved or suspended in an aqueous vehicle with similar functional excipients as the capsule (including a high-HLB surfactant and an organic acid, together with a polysaccharide such as maltodextrin). Prior to administration, the vial was shaken to ensure uniform dispersion. The liquid is essentially a ready-to-use nanoemulsion of CBD, designed for rapid absorption through the gastrointestinal mucosa upon swallowing.

#### Reference product

A 40 mg CBD isolate capsule (prepared from the same CBD isolate batch, filled as a powder into a gelatin capsule with no bioavailability enhancers, only MCC as excipient). This served as the control, representing standard pure CBD with no specialized formulation. The reference capsule contained only the CBD isolate and an inert pharmaceutical filler (no surfactants or other solubilizing excipients). All three products were manufactured under GMP conditions by Imuneks Farma and had matching CBD content verified by assay.

In each treatment period, after an overnight fast of ≥ 10 h, subjects consumed a standardized low-calorie, low-fat breakfast approximately 30 min before dosing. The meal provided roughly 300–350 kcal with < 10 g of fat (for example, toast with jelly and juice) in order to achieve “fed” status while minimizing the enhancement of CBD absorption that a high-fat meal would produce. (Taylor et al. 2018) Dosing occurred at a consistent morning time (~ 8 AM) under supervision. For the capsule formulations (Test-1 and Reference), subjects swallowed the capsule with 240 mL of water while seated. For the liquid (Test-2), the full 10 mL vial content was ingested orally; the vial was then rinsed three times with portions of the 240 mL of water, which were also swallowed to ensure no residual CBD remained in the vial or oral cavity. (FDA 2003) Subjects were instructed to briefly swish and then swallow during rinses to minimize any CBD sticking to the oral mucosa. Immediately after dosing, an empty-mouth check was performed to confirm the dose was fully consumed. Subjects remained upright (seated or standing) for at least 4 h post-dose (lying on the right side was permitted in case of dizziness). Water was restricted from 1 h pre-dose until 2 h post-dose (aside from the 240 mL given at dosing); thereafter water was allowed ad libitum. No food was allowed until 6 h post-dose, after which a standardized lunch and snacks were provided at scheduled times. Caffeine, alcohol, grapefruit juice, and strenuous activity were prohibited during each study period. Compliance with all procedures was excellent, and no subject vomited or lost their dose during the study.

### Blood sample collection

During each treatment period, blood samples for PK analysis were collected at pre-dose (− 1 h) and at 0.133 h (8 min), 0.25, 0.5, 0.75, 1.0, 1.5, 2.0, 2.5, 3.0, 3.5, 4.0, 4.5, 5.0, 6.0, 8.0, 12.0, 23.0, 48.0, and 72.0 h after dosing. This intensive sampling schedule provided very frequent early time points to precisely define the absorption phase and capture C_max, and extended out to 72 h to characterize the elimination phase. The 72-h duration was considered sufficient to cover over three CBD half-lives (human t_1/2 ~ 24–36 h), ensuring that > 90% of the total AUC was captured. The 23 h sample was included to approximate a 24 h level before the next day, and a 48-h sample to help define the terminal elimination slope. Blood (~ 8 mL per time point) was drawn from an indwelling catheter or via direct venipuncture into K_2 EDTA tubes, under minimal light (sodium vapor lamp) to prevent potential degradation of cannabinoids by UV exposure. Samples were kept on ice and centrifuged within 30 min at 4 °C to isolate plasma, which was then stored at − 80 °C until analysis.

In addition to the PK samples, plasma cortisol was measured as a pharmacodynamic endpoint. Blood for cortisol was collected at 08:00 h (pre-dose), at 10:00, 12:00 and 16:00 h (2.0-, 4.0-, and 8.0-hours post-dose in each period) These times were chosen to capture potential acute changes in cortisol (which has a diurnal peak in the early morning and declines through midday). The cortisol measurements were intended to explore whether CBD acutely alters stress hormone levels in this healthy setting, given some evidence of CBD’s anxiolytic effects in stressful conditions (e.g., public speaking tests). (Appiah-Kusi et al. 2020)

### Bioanalytical methods

Plasma concentrations of CBD and its primary metabolite 7-hydroxy-CBD (7-OH-CBD) were quantified by a validated liquid chromatography–tandem mass spectrometry (LC–MS/MS) method, which was demonstrated to be sensitive, rapid, reproducible and accurate enough to apply to the determination of Cannabidiol in human plasma over a range of (0.100-9.973) ng/ml for bioequivalence studies purposes. Sample preparation involved liquid–liquid extraction with tert-butyl methyl ether, which has been shown to efficiently recover cannabinoids from plasma. Chromatography was performed on a Phenomenex Gemini C18 column (50 × 3.0 mm, 3 μm particle size) with a mobile phase consisting of 55% acetonitrile, 15% methanol, and 30% 0.005 M ammonium formate buffer (pH ~ 5), at a flow rate of 0.4 mL/min. CBD and 7-OH-CBD were detected by tandem MS in positive electrospray ionization mode, monitoring their specific precursor → product ion transitions. The assay was linear over a concentration range of 0.100 to 10.0 ng/mL for CBD (and a similar range for 7-OH-CBD). The lower limit of quantification (LLOQ) for CBD was 0.1 ng/mL. Assay precision and accuracy met bioanalytical acceptance criteria (inter-day precision CV 3–7% and accuracy 100–108% at quality control levels). For 7-OH-CBD, calibration and validation parameters were likewise within acceptable ranges. No significant matrix effects or interference were observed, and incurred sample re-analysis showed > 98% of re-assayed samples were within 20% of the original value, confirming assay reliability.

Plasma cortisol was measured using a standard automated chemiluminescent immunoassay in the hospital’s clinical laboratory. The normal reference range for morning cortisol in this lab is approximately 5–25 µg/dL. Quality control samples for cortisol were within expected ranges throughout the study.

### Pharmacokinetic and statistical analysis

Pharmacokinetic parameters for CBD and 7-OH-CBD were calculated by standard non-compartmental methods using Phoenix WinNonlin^®^ (v8.3, Certara) and SAS^®^ (v9.4, SAS Institute). The maximum observed plasma concentration (C_max) and time to reach C_max (tmax) were obtained directly from the concentration–time data. The area under the plasma concentration–time curve (AUC) was calculated using the linear trapezoidal rule. We determined AUC from time zero to the last measurable concentration (AUC_0–t, where t = 72 h for CBD in most cases) and extrapolated to infinity (AUC_0–∞) when data permitted. Because quantifiable concentrations of CBD were observed through 72 h for all formulations, AUC_0–72 was used as the primary exposure metric for comparison across treatments. In all subjects, > 90% of the total AUC was covered by the 72-h sampling interval (extrapolated < 10%), indicating that the 72-h duration was sufficient and justifying the use of AUC_0–72 for bioavailability comparisons. This satisfies the usual regulatory criterion that ≥ 80% of the AUC be captured by the last time point. The apparent terminal elimination half-life (t_1/2) of CBD was estimated by log-linear regression of the terminal phase of the concentration–time curve (using at least three data points in the log-linear decline, typically from ~ 12 h to 72 h for CBD). For 7-OH-CBD, analogous parameters (C_max, tmax, AUC_0–72, t_1/2) were determined.

Descriptive statistics (mean, standard deviation (SD), median, range, and coefficient of variation (CV%)) were calculated for plasma concentrations and PK parameters of CBD and 7-OH-CBD for each formulation. tmax values were treated as ordinal data and summarized as medians (with ranges); formulation tmax values were compared using Friedman’s non-parametric test (with post-hoc Wilcoxon signed-rank tests for pairwise comparisons as needed). For C_max and AUC, a mixed-effects analysis of variance (ANOVA) appropriate for the 3 × 3 crossover design was used. The ANOVA model included fixed effects of treatment, period, and sequence, and a random effect of subject nested within sequence. Period and sequence effects were tested to check for any carryover or time-period effects. There were no significant period or sequence effects for any PK endpoint (*p* > 0.5), confirming adequate washout and no systematic time trends.

To compare relative bioavailability, geometric least-squares mean ratios (Test/Reference) for C_max and AUC were computed for each test product versus the reference, along with their 90% confidence intervals (CIs). These ratios were derived from the ANOVA on log-transformed values. Although our objective was to characterize relative bioavailability rather than to demonstrate bioequivalence, the 90% CIs provide a measure of precision and were used to assess if the differences between formulations were statistically significant; in a bioequivalence framework, lack of overlap with the 80–125% equivalence range would indicate non-equivalence. (EMA 2010) We also calculated the ratio of Test 1 vs. Test 2 in a similar manner (to explore differences between the two novel formulations). Additionally, an equivalence test for Test 1 vs. Reference and Test 2 vs. Reference was pre-specified using the conventional 80–125% acceptance range. As expected, the resulting 90% CIs lay entirely outside 80–125%, indicating that the test products were not bioequivalent to the reference but rather produced far higher exposure.

All statistical tests were two-sided, with *p* < 0.05 considered statistically significant. Safety data were summarized descriptively, and the incidence of adverse events was tabulated by treatment. No formal statistical comparisons of safety endpoints between treatments were planned due to the small sample size. The study conduct and reporting followed CONSORT guidelines for transparency. (Schulz et al. 2010). The statistical analysis plan was finalized before database lock and no unplanned analyses were introduced.

Safety assessments in this study included physical examinations, vital signs (blood pressure, heart rate, temperature) at screening and various post-dose time points, 12-lead electrocardiograms (ECGs), and a panel of clinical laboratory panel (hematology, electrolytes, renal function, hepatic panel — ALT, AST, ALP, GGT, bilirubin — and fasting glucose/lipids) were performed. Harms were assessed by a blinded physician-investigator at 2, 8, 24 and 72 h post-dose and at follow-up. Adverse events were monitored throughout the study and were coded for severity and relationship to the study drug. All safety findings were reviewed.

## Results

### Participant disposition and baseline characteristics

Twelve male volunteers were enrolled and received at least one dose of study medication. Nine subjects completed all three treatment periods and were included in the PK analysis. Three subjects were withdrawn from the study as described above: two subjects had unexpected positive urine cannabinoid tests at the start of Period 2 (indicating undisclosed cannabis use between study periods) and were withdrawn, and one subject developed a moderate adverse event (a transient febrile viral illness) before Period 3 and was withdrawn as a precaution.

The demographic characteristics of the nine completing subjects were as follows: mean age 29 years (range 19–44), mean weight 74 kg (range 65–82), and all were non-smokers with no significant medical history. Baseline laboratory values and vital signs were within normal limits for all subjects. Compliance with the protocol’s dietary and activity restrictions was high (aside from the aforementioned individuals who were withdrawn for protocol violations). All nine completers were included in the safety analyses for the treatments they received (in total, 11 subjects contributed safety data across the study).

### Pharmacokinetic profiles of CBD

**Fig. 1:**
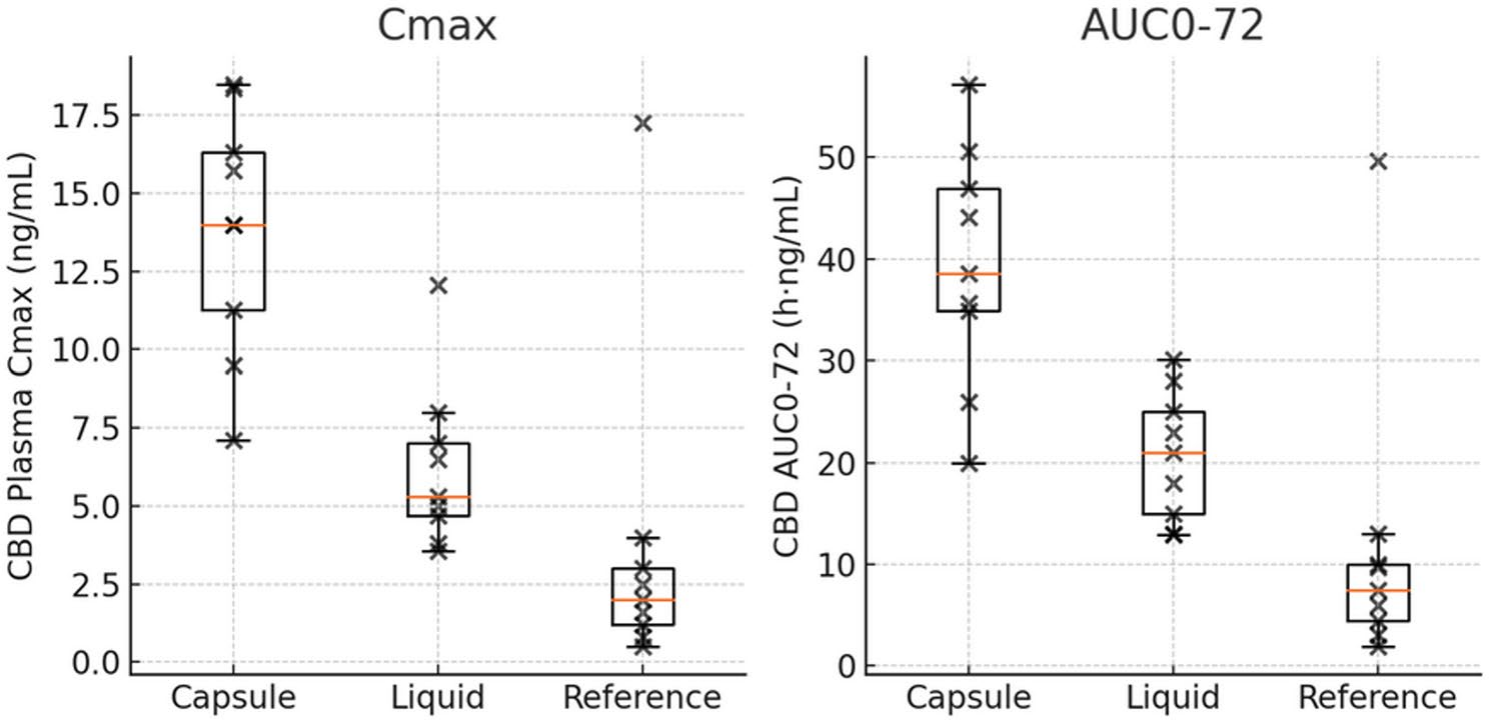
Comparison of CBD peak concentrations C_max, and total exposures AUC_0–72). Comparison of CBD peak concentration (C_max) and total exposure (AUC_0–72) for each formulation (*N* = 9). Data are shown as box-and-whisker plots with individual values (●). The enhanced capsule (Test-1) achieved by far the highest C_max (median ~ 14 ng/mL) and AUC_0–72 (~ 38 h·ng/mL), while the unformulated reference capsule had the lowest (median C_max ~ 2 ng/mL, AUC_0–72 ~ 12 h·ng/mL). The enhanced liquid (Test-2) was intermediate (median C_max ~ 6 ng/mL, AUC_0–72 ~ 20 h·ng/mL). Inter-subject variability in C_max and AUC was markedly lower for the two enhanced formulations (narrower boxes and shorter whiskers) than for the reference, indicating more consistent absorption with the novel formulations

All formulations resulted in quantifiable plasma CBD concentrations through the 72-hour post-dose period in every subject. Table 1 summarizes the principal pharmacokinetic parameters for CBD for each formulation. In brief, the enhanced formulations resulted in substantially higher CBD exposure and faster absorption compared to the unformulated reference. The enhanced capsule (Test-1) achieved a mean C_max of 14.1 ng/mL and AUC_0–72 of 38.0 h·ng/mL, whereas the enhanced liquid (Test-2) showed a mean C_max of 6.20 ng/mL and AUC_0–72 of 20.2 h·ng/mL. By contrast, the reference 40 mg CBD isolate capsule produced a much lower mean C_max of 2.44 ng/mL and AUC_0–72 of 11.7 h·ng/mL. The time to reach C_max (median tmax) was 1.0 h for the liquid, 2.0 h for the enhanced capsule, and 6.0 h for the reference (Table 1; Fig. 1). Notably, inter-individual variability in these parameters was greatly reduced with the new formulations: for example, the coefficient of variation in C_max was approximately 27% for the capsule and 44% for the liquid, compared to 134% for the reference capsule (Table 1).

**Table 1:** Pharmacokinetic parameters of CBD (parent compound) after a single 40 mg oral dose of each formulation (*N* = 9, fed conditions)

Parameter	CBDNEXT Supra Capsule (40 mg)	CBDNEXT Supra Liquid (40 mg)	Reference Capsule (40 mg)
C_max (ng/mL), mean ± SD	14.1 ± 3.9	6.20 ± 2.7	2.44 ± 3.3
tmax (h), median [range]	2.0 [1.0–4.0]	1.0 [0.75–2.5]	6.0 [4.0–8.0]
AUC_0–72 (h·ng/mL), mean ± SD	38.0 ± 10.0	20.2 ± 6.5	11.7 ± 10.7
t_1/2 (h), mean ± SD	25.4 ± 6.2	24.1 ± 5.6	22.3 ± 7.0

SD, standard deviation; C_max, maximum observed plasma concentration; tmax, time of C_max; AUC_0–72, area under the plasma concentration–time curve from 0 to 72 h; t_1/2, terminal elimination half-life

Geometric CV% for C_max: 27.5% (capsule), 43.9% (liquid), 134.1% (reference); for AUC_0–72: 26.3%, 32.0%, 91.3%, respectively

Statistical analysis confirmed that both novel formulations provided significantly greater CBD exposure than the reference. The enhanced capsule yielded a ~ 5.66-fold higher C_max and ~ 3.28-fold higher AUC_0–72 than the reference (geometric mean ratios ~ 566% and ~ 328%, *p* < 0.001 for both), and the enhanced liquid yielded ~ 2.48-fold higher C_max and ~ 1.66-fold higher AUC_0–72 than the reference (GMR ~ 248% and ~ 166%, *p* < 0.001). The direct comparison of the two test formulations indicated that the capsule achieved higher exposure than the liquid (approximately 2.29-fold higher C_max and 1.98-fold higher AUC_0–72; *p* = 0.002). These results demonstrate a clear rank order of bioavailability: Capsule > Liquid > > Reference.

One participant in the reference product group exhibited plasma CBD levels approximately sevenfold higher than the group median. This outlier may be attributed to several factors including interindividual variability in CBD metabolism, potential differences in gastric emptying, adherence to dosing instructions, or metabolic polymorphisms affecting drug disposition. Such variability is not uncommon in pharmacokinetic studies and reflects the inherent biological diversity in drug absorption and metabolism among individuals.

The primary phase I metabolite, 7-OH-CBD, reached only a small fraction of the parent CBD levels. Across treatments, mean 7-OH-CBD C_max values were 1.8 ng/mL for the enhanced capsule, 1.0 ng/mL for the enhanced liquid, and 0.4 ng/mL for the reference, corresponding to roughly 10–13% of the parent C_max in each case. The metabolite AUC_0–72 values were similarly low (8.0, 4.5, and 1.8 h·ng/mL, respectively, for capsule, liquid, and reference; ~21–22% of parent AUC for the tests vs. ~15% for reference). The 7-OH-CBD tmax tended to lag behind CBD by about 0.5–2 h (median metabolite tmax ~ 3 h for capsule, 2 h for liquid, 6 h for reference). The 7-OH-CBD elimination phase appeared to parallel that of CBD, with an estimated t_1/2 of approximately 20–26 h (though the terminal phase for 7-OH-CBD was less well-characterized due to low concentrations by 48–72 h). No other major circulating metabolites (e.g., 7-carboxy-CBD) were detected above the assay threshold.

**Fig. 2:**
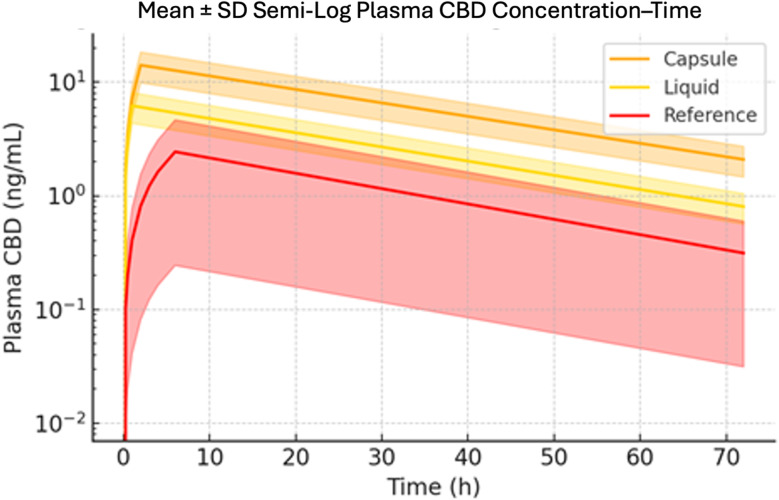
Mean ± SD Semi-Log Plasma CBD Concentration–Time Profiles (0–72 h). Mean plasma CBD concentrations for the CBDNEXT Supra capsule, CBDNEXT Supra liquid, and unformulated reference capsule are plotted on a logarithmic y-axis (*N* = 9). Shaded ribbons represent ± 1 SD at each time-point, illustrating inter-subject variability. Both novel formulations maintain markedly higher concentrations throughout the 72 h sampling window, while all three curves exhibit a parallel terminal decline consistent with similar elimination half-lives

**Fig. 3:**
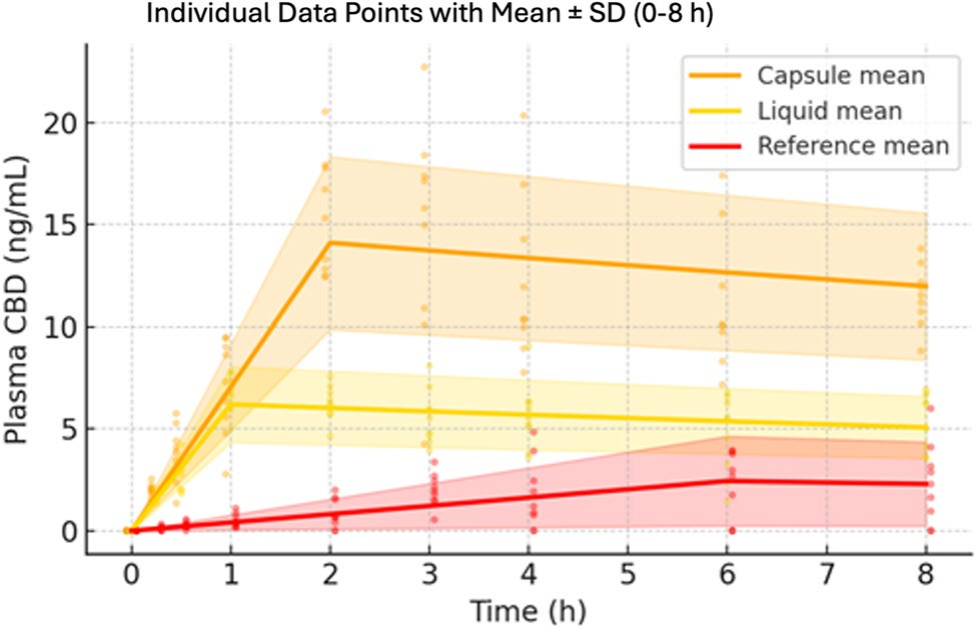
Early absorption phase with individual data (0–8 h). Dots show each volunteer’s plasma CBD concentration at every sampling time (*N* = 9 per formulation); bold lines depict the formulation means and shaded ribbons ± SD. The liquid reaches peak levels fastest (~ 1 h), the capsule attains the highest Cmax (~ 14 ng/mL at 2 h), and the reference capsule displays the slowest and lowest absorption. The overlay of individual points demonstrates reduced variability for the enhanced formulations compared with the unformulated reference

Mean plasma concentration–time profiles of CBD for the three formulations are presented in Figs. 2 and 3. Figure 2 shows the plasma CBD profiles on a semi-logarithmic scale over the entire 72-hour observation period, and Fig. 3 focuses on the initial 0–8 h absorption phase on a linear scale. These profiles highlight the dramatically different absorption kinetics and extents of exposure among the capsule, liquid, and reference formulations.

Consistent with the profile data, the elimination phase of CBD appeared similar for all three formulations. Once absorbed, CBD exhibited an apparent terminal half-life on the order of 22–26 h for capsule, liquid, and reference alike (Table 1). One subject displayed a reference-product Cmax of 5.0 ng/mL (~ 7-fold the group median); review of dosing records, meal timing, and assay QC revealed no error, and the value was retained as legitimate inter-individual variability. The formulation differences were confined to the absorption phase and extent, with no indication of any alteration in elimination kinetics.

The statistical comparisons further underline these differences in bioavailability (Table 2). For Test-1 (capsule) vs. Reference, the geometric mean C_max ratio was 566% (90% CI: 356–900%), and the geometric mean AUC_0–72 ratio was 328% (90% CI: 226–478%). In both cases, the lower bound of the 90% CI was well above 125%, confirming a highly significant increase (*p* < 0.001) in CBD exposure with the enhanced capsule. For Test-2 (liquid) vs. Reference, the C_max ratio was 248% (90% CI: 156–394%) and the AUC_0–72 ratio was 166% (90% CI: 114–241%), also statistically significant increases (*p* < 0.001). By contrast, comparing Test-1 vs. Test-2 showed that the capsule had ~ 2.29-fold higher C_max than the liquid (90% CI: 140–374%) and ~ 1.98-fold higher AUC (90% CI: 136–288%), a difference that was significant (*p* = 0.002) despite the small sample size. Although not powered to definitively compare the two novel formulations, this trend suggests the solid capsule achieved greater systemic exposure than the liquid for the same 40 mg CBD dose. Both test formulations were vastly higher in exposure than the reference, which performed poorly as anticipated for an unenhanced CBD product.

**Table 2:** Geometric mean ratios (GMR) of CBD c_max and AUC_0–72 for pairwise comparisons between formulations

Comparison	C_max GMR (%) (90% CI)	AUC_0–72 GMR (%) (90% CI)	*p*-value^1^
CBDNEXT Supra Capsule vs. Reference	566% (356–900%)	328% (226–478%)	< 0.001
CBDNEXT Supra Liquid vs. Reference	248% (156–394%)	166% (114–241%)	< 0.001
CBDNEXT Supra Capsule vs. Supra Liquid	229% (140–374%)	198% (136–288%)	0.002

CI: confidence interval

^1^Mixed-effects ANOVA on log-transformed values (two-sided α = 0.05; *N* = 9)

### 7-Hydroxy-CBD metabolite kinetics

The primary Phase I metabolite of CBD, 7-hydroxy-cannabidiol (7-OH-CBD), was also measured in plasma. In all subjects and for all formulations, 7-OH-CBD concentrations were substantially lower than parent CBD concentrations. This metabolite is formed via CYP-mediated oxidation of CBD in the liver. Table 3 presents the comparative PK parameters for 7-OH-CBD. The mean plasma 7-OH-CBD peak concentrations (C_max) were 1.8 ng/mL for the capsule, 1.0 ng/mL for the liquid, and 0.4 ng/mL for the reference. Thus, for each treatment, the metabolite C_max was only about 10% (capsule) to 13% (liquid) of the parent C_max (for example, 1.8 vs. 14.1 ng/mL for capsule). A similar rank order was observed as with the parent compound: the enhanced capsule produced the highest 7-OH-CBD levels, the liquid intermediate, and the reference the lowest, reflecting the greater amount of CBD available for metabolism with the test products. However, the metabolite-to-parent exposure ratio was slightly lower for the test formulations than for the reference. The AUC_0–72 of 7-OH-CBD was 8.0 h·ng/mL for the capsule, 4.5 h·ng/mL for the liquid, and 1.8 h·ng/mL for the reference. As a percentage of parent AUC, these values are approximately 21% (capsule), 22% (liquid), and 15% (reference). This suggests that a somewhat greater fraction of absorbed CBD was metabolized to 7-OH-CBD with the enhanced formulations compared to the reference, which could be due to saturation of downstream metabolism at the lower exposures from the reference (though at a single 40 mg dose, major saturation of CBD metabolism is not expected). In any case, 7-OH-CBD exposures were an order of magnitude lower than parent CBD for all treatments. The tmax of 7-OH-CBD tended to lag behind the parent by about 0.5 to 2 h. Median tmax for 7-OH-CBD was ~ 3 h for the capsule, 2 h for the liquid, and 6 h for the reference, a slight delay consistent with formation-time lag (i.e., CBD must first be absorbed and metabolized to 7-OH). By 8 h post-dose, 7-OH-CBD levels had declined in parallel with CBD. The terminal half-life of 7-OH-CBD appeared similar to or slightly shorter than that of CBD (estimated ~ 20 h), although quantitation beyond 24–48 h was limited by the low levels.

No unusual or unexpected metabolites (such as 7-carboxy-CBD) were detected in plasma in this study. As expected, 7-OH-CBD was the predominant circulating CBD metabolite observed.

These metabolite data indicate that the enhanced formulations, by delivering more CBD into the systemic circulation, also resulted in greater absolute formation of 7-OH-CBD. However, the metabolic conversion as a fraction of absorbed dose did not increase proportionally, since the parent exposure increased more than the metabolite exposure. This could imply a partial saturation of first-pass metabolism at the higher concentrations achieved by the test products, or simply that 7-OH-CBD is rapidly further metabolized to subsequent metabolites (e.g., glucuronides). In any case, the presence of higher 7-OH-CBD levels with the test formulations is consistent with their higher bioavailability of CBD. Importantly, 7-OH-CBD itself is not known to have significant independent pharmacological activity (in contrast to THC’s active 11-hydroxy metabolite); CBD’s activity is mainly attributed to the parent compound. Thus, the differences in metabolite levels are unlikely to have direct clinical significance, except as a marker of the extent of first-pass metabolism. (Bergamaschi et al. 2011)

Table 3 summarizes the relative bioavailability results for the two CBDNEXT Supra test products versus the unformulated reference, as well as the head-to-head comparison between the two test formulations. All values are expressed as geometric mean ratios (GMRs) of the pharmacokinetic parameters for the row comparator relative to the column comparator, with 90% CIs. Key findings include:

- *Capsule vs. Reference*: The enhanced capsule delivered **566%** of the reference’s C_max (≈ 5.7-fold higher) and **328%** of the reference’s AUC_0–72 (≈ 3.3-fold higher). The 90% CIs for both C_max and AUC lie entirely above 125%, confirming that the capsule produced a statistically significant and clinically meaningful increase in CBD exposure (*p* < 0.001).
- *Liquid vs. Reference*: The enhanced liquid provided **248%** of the reference’s C_max (≈ 2.5-fold higher) and **166%** of the reference’s AUC_0–72 (≈ 1.7-fold higher). Here too, the 90% CIs are above 125%, indicating a significant increase in exposure (*p* < 0.001) with the liquid formulation.
- *Capsule vs. Liquid*: The capsule achieved **229%** of the liquid’s C_max (≈ 2.3-fold higher) and **198%** of the liquid’s AUC_0–72 (≈ 2.0-fold higher). The capsule thus outperformed the liquid in both peak and overall exposure to CBD (*p* = 0.002), despite both being enhanced formulations.

Because all confidence intervals for Test vs. Reference comparisons are well outside the conventional 80–125% equivalence range, neither test product is bioequivalent to the reference—in fact, both greatly exceed the reference in systemic exposure. Likewise, the capsule vs. liquid comparison shows 90% CIs that do not include 100%, indicating a significant difference between the two enhanced formulations (with the capsule > liquid in exposure). These findings confirm that the formulation technology markedly increased CBD bioavailability.

**Table 3:** Pharmacokinetic parameters for 7-hydroxy-CBD after a single 40 mg dose (*N* = 9)

Parameter	CBDNEXT Supra Capsule	CBDNEXT Supra Liquid	Reference Capsule
C_max (ng/mL), mean ± SD	1.8 ± 0.8	1.0 ± 0.5	0.4 ± 0.2
tmax (h), median [range]	3 [2–4]	2 [1–4]	6 [4–12]
AUC_0–72 (h·ng/mL), mean ± SD	8.0 ± 4.0	4.5 ± 2.2	1.8 ± 0.9
t_1/2 (h), mean ± SD	26.3 ± 6.0	24.0 ± 5.2	20.1 ± 4.4

Note: 7-OH-CBD = 7-hydroxy-cannabidiol. Due to the low concentrations of this metabolite, there is greater analytical variability. The half-life (t_1/2) was estimated for each subject from the terminal log-linear phase (≥ 3 data points between 8 h and 72 h) and is reported as the mean ± SD

### Safety and tolerability

All 11 dosed subjects contributed to the safety set (3 × capsule doses, 4 × liquid, 4 × reference). Treatment-related AEs (headache) occurred in 1/11 (enhanced capsule), 2/11 (enhanced liquid), 1/11 (reference). All treatments were generally well tolerated. No serious adverse events (SAEs) or severe AEs were observed during the study. A total of four AEs were reported. The only AE considered possibly related to the study drugs was headache, which occurred in 4 out of 11 subjects who received at least one dose (36%). Specifically, one subject reported a moderate tension-type headache on the evening after receiving the enhanced capsule; two subjects reported mild headaches on the day they received the enhanced liquid; and one subject reported a mild headache on the day of the reference capsule. All headache events were transient and resolved spontaneously within 4–12 h or with minimal analgesic treatment (e.g., a single dose of paracetamol); all subjects continued in the study (the one subject with a moderate headache had already completed all periods). There were no patterns suggesting that one formulation caused more headaches than another, given the small numbers (1 case with the capsule, 2 with the liquid, 1 with the reference). It is worth noting that mild, transient headache is a commonly reported side effect in clinical studies, and in this trial, it may also have been influenced by factors such as overnight fasting or caffeine withdrawal prior to dosing.

No other treatment-related side effects (such as dizziness, somnolence, or gastrointestinal upset) were reported during any of the confinement periods. In particular, no subject reported feeling intoxicated or “high” at any time, which is consistent with CBD’s non-psychoactive profile and the absence of THC in all formulations. There were no instances of euphoria, dysphoria, or sedation beyond placebo-like levels, and neurological examinations remained normal throughout. Additionally, no clinically significant changes were noted on 12-lead ECGs obtained at screening and end-of-study– heart rhythm and conduction parameters remained stable in all subjects.

Laboratory evaluations revealed no clinically significant abnormalities. In particular, liver enzymes (ALT, AST, ALP, GGT) and total bilirubin did not show any notable post-dose elevations for any treatment. The mean changes in hepatic markers from baseline were < 5%, and all individual values remained within normal reference ranges throughout the study. Renal function markers (BUN, creatinine) likewise remained within normal limits in all subjects. Hematology values (complete blood count) showed no meaningful changes after dosing.

Vital signs (blood pressure and heart rate) measured periodically after dosing showed no treatment-related trends. A slight, transient decrease in blood pressure (~ 5 mmHg systolic on average) was observed in a few subjects following CBD dosing, but this occurred in all groups including the reference and was within normal physiologic fluctuations. This small drop in blood pressure is consistent with CBD’s known mild vasodilatory effects, but it was not clinically significant. There were no episodes of orthostatic hypotension, syncope, or clinically significant changes in heart rate. All recorded blood pressure and pulse values remained in the normal range (See Supplementary Table S1 for individual data).

Plasma cortisol levels remained within normal physiological ranges at all measured time points for all treatments. The mean baseline (pre-dose) cortisol was ~ 14 µg/dL at 8 AM (morning), and by 4 h post-dose (around noon) the mean cortisol declined to ~ 8 µg/dL, reaching ~ 6 µg/dL by 8 h, consistent with the normal diurnal rhythm (cortisol naturally peaks in the early morning and falls by afternoon). (Weitzman et al. 1971) Importantly, there were no significant differences in cortisol profiles between the enhanced capsule, enhanced liquid, and reference. At 2 h, 4 h, and 8 h post-dose, cortisol values were statistically equivalent across treatments (ANOVA *p* > 0.7 at each time point). Thus, acute CBD administration (40 mg), even with enhanced bioavailability, did not measurably alter cortisol secretion relative to baseline or relative to the control. The small reductions in cortisol observed over time are attributable to time-of-day effects rather than the drug. This suggests that in healthy, unstressed volunteers, a single dose of CBD– even when achieving higher plasma levels– does not acutely suppress hypothalamic–pituitary–adrenal (HPA) axis activity. All cortisol readings at 8 h remained well within the normal range for afternoon cortisol (typically 3–10 µg/dL). These findings are noteworthy given preclinical evidence that CBD may attenuate stress-induced cortisol spikes; in our non-stressed study setting, no such effect was apparent, as expected.

No subject showed any signs of cannabinoid intoxication or psychoactive effects at any point, which is consistent with the known profile of pure CBD and the lack of THC in these products. There were no reports of feeling “stoned” or any hallucinations or dysphoric symptoms. Subjects’ alertness and cognition, as assessed informally during clinical observations, remained normal after all doses.

In summary, both the CBDNEXT Supra capsule and liquid were as well tolerated as the pure CBD isolate. The safety profile observed in this study aligns with the literature on CBD in humans, which indicates that CBD is generally well tolerated even at doses much higher than 40 mg, with a low incidence of serious adverse effects. The mild headaches reported in our trial resolved quickly and did not show a consistent recurrence with any particular formulation. There were no adverse events related to laboratory abnormalities. No subject withdrew due to a safety issue related to the study drug (the one withdrawal due to an adverse event was for an intercurrent infection unrelated to dosing). Overall, the results raise no new safety concerns for the enhanced formulations. If anything, the reduced variability in CBD levels with the new formulations could translate into more predictable dosing and fewer unexpected pharmacodynamic fluctuations, thereby maintaining a wide safety margin.

## Discussion

### Enhanced absorption and onset

This randomized crossover study demonstrated that the novel CBDNEXT Supra oral formulations (both capsule and liquid) substantially enhance the bioavailability of CBD compared to the reference poduct (a standard CBD isolate capsule), even under conditions deliberately not optimized for absorption (i.e., with only a low-fat meal). The enhanced capsule in particular achieved dramatically higher peak plasma CBD concentrations and AUC than the reference, along with markedly lower inter-subject variability. The enhanced liquid also showed significantly improved exposure and the fastest absorption. To our knowledge, this is one of the first head-to-head comparisons of advanced CBD formulations in humans using a moderate 40 mg dose under low-fat fed conditions. The findings have several important implications.

The CBDNEXT Supra capsule achieved a C_max of ~ 14 ng/mL at ~ 2 h, versus only ~ 2.4 ng/mL at ~ 6 h for the unformulated CBD. This ~ 6-fold higher C_max represents a 566% increase in peak exposure, while AUC_0–72 was ~ 3.3-fold higher (328% increase) relative to the reference. These improvements are striking and indicate a major enhancement in the fraction of CBD absorbed. In practical terms, the 40 mg Supra capsule delivered as much or more CBD into the bloodstream as an oral dose of roughly 150–200 mg of pure CBD would have under the same low-fat conditions (based on the reference capsule’s exposure). The Supra liquid, while not as high as the capsule, still achieved a ~ 2.5-fold higher C_max and ~ 1.7-fold higher AUC than the reference. Notably, the liquid’s absorption was extremely rapid (tmax ~ 1.2 h), meaning it could reach effective plasma concentrations more quickly, whereas the capsule’s tmax (2 h) was slightly slower but still much earlier than the reference (5–6 h). This suggests a potential differentiation in use: the liquid provides a faster onset of action, whereas the capsule maximizes total exposure. Both formulations, however, achieved substantially faster and greater absorption than typically reported for oral CBD. In published studies with similar doses of generic CBD, tmax values often range 3–5 h in fasted conditions, and even longer (5–6 + h) with plain capsule forms taken with food. (Millar et al. 2018) In our study, the low-fat meal may have slightly delayed the reference formulation’s absorption (to 5–6 h), but the new formulations overcame that delay, likely by facilitating rapid CBD dissolution and uptake in the proximal gut. These dose-normalised comparisons assume linear pharmacokinetics across the 30–50 mg range, an assumption supported by published single-dose studies (Millar et al. 2018) but not formally demonstrated for every formulation. Moreover, differences in fed versus fasted conditions add variability; CBD exposure typically rises two- to four-fold between fasted and high-fat states, so cross-study extrapolations must be viewed as exploratory only.

### Reduced variability with new formulations

The greatly reduced inter-subject variability observed with the new formulations is an especially important finding. High variability has long plagued oral CBD, making it difficult to predict patient exposure and clinical response. In this study, the reference isolate capsule showed a > 90% CV in AUC and > 130% in C_max, indicating that some individuals absorbed almost no CBD while others achieved modest levels. In contrast, the enhanced capsule had CVs on the order of 26–28%, akin to the variability seen with many conventional pharmaceuticals, and the enhanced liquid CVs were only slightly higher (~ 32–44%)– still far lower than the reference. This consistency likely reflects the more reliable solubilization of CBD provided by the surfactant and other excipients. Each subject’s gastrointestinal environment can vary (pH, bile salt secretion, gastric emptying, etc.), and an unformulated hydrophobic drug like CBD may precipitate or pass through unabsorbed in many cases. The Supra formulations appear to buffer against those individual differences by maintaining CBD in a soluble, bioaccessible form across individuals. This improved uniformity is highly desirable clinically, as it allows more reliable dosing to achieve target concentrations and could lead to more predictable therapeutic outcomes. In practice, every subject in our study showed higher CBD exposure on the test products than on the reference. For instance, individual C_max values on the reference ranged from only ~ 0.5 ng/mL in one subject up to ~ 5 ng/mL in another, whereas on the enhanced capsule, all subjects had C_max between ~ 9 and 18 ng/mL. Achieving more consistent plasma levels can make it easier for prescribers to predict dosing requirements and for patients to receive the intended effect without excessive trial-and-error.

### Mechanism of improved bioavailability

The dramatic increase in CBD exposure with the Supra formulations can be attributed to their specialized composition and mode of action. Both the capsule and liquid contain CBD in combination with a high-HLB emulsifier (e.g., polysorbate 80) and an organic acid (e.g., stearic or citric acid), among other excipients. Upon ingestion, the surfactant likely self-emulsifies with gastrointestinal fluids, creating micro- or nano-emulsions of CBD. The presence of an acid could assist by providing an acidic micro-environment that keeps CBD in a more soluble form (since CBD is a weakly basic compound), or possibly by affecting intestinal paracellular permeability (some medium-chain fatty acids like capric/stearic acid can transiently enhance absorption by loosening tight junctions). The patent underlying these formulations reported that combining an acid with the surfactant along with a silica and dextrin compound, yielded a surprisingly large enhancement in the dissolution and absorption of cannabinoids. Additionally, the capsule’s solid matrix (including maltodextrin and colloidal silica) may serve to disperse CBD at a molecular level and prevent aggregation or recrystallization during transit through the GI tract. The net result is that a much larger fraction of the 40 mg dose is dissolved and presented to the intestinal membrane for absorption, even in the absence of dietary lipids.

Moreover, surfactants like polysorbate 80 can increase intestinal membrane fluidity and may inhibit P-glycoprotein or other efflux transporters, further boosting absorption by reducing CBD efflux back into the gut lumen. (Yuan et al. 2007) It is also possible that the emulsified CBD is partly absorbed via the intestinal lymphatic pathway (as is known for highly lipophilic compounds). If a portion of CBD is transported through intestinal lymph (chylomicron-associated absorption), it would bypass first-pass hepatic metabolism and lead to higher systemic bioavailability. The inclusion of lipidic excipients in the formulation (even though in solid form for the capsule) could favor such lymphatic uptake. (Caliph et al. 2000) We did not directly assess lymphatic absorption, but the observation of significantly higher 7-OH-CBD levels with the test products suggests that first-pass metabolism was still substantial (since 7-OH-CBD formation occurs in the liver). However, the fact that the metabolite-to-parent ratio did not increase in proportion to the parent implies that some portion of CBD might have been absorbed via non-portal routes or that the liver’s metabolic capacity was not linearly challenged at the higher exposures. (Zgair et al. 2017) Overall, our findings provide real-world validation of the patented formulation approach used in the CBDNEXT Supra products, demonstrating superior performance compared to a plain isolate capsule under conditions where ordinarily CBD absorption would be low. The substantial increase in systemic exposure achieved by these excipient-based formulations shows that relatively simple ingredients (surfactants + acids + dispersion matrix) can rival more complex delivery systems in enhancing oral CBD bioavailability. (Hegde et al. 2022)

### Comparison to other studies

It is useful to contextualize our results with other published pharmacokinetic studies of oral CBD. (Williams et al. 2021) conducted a randomized crossover study in 15 healthy subjects comparing five different oral CBD preparations standardized to a 30 mg dose. They tested a CBD oil tincture, a water-soluble CBD powder drink, a 20% CBD in lipid solution, a 5% CBD concentration liquid, and a 5% CBD concentration powder. In that study, the best-performing formulation was a 5% CBD liquid (essentially a nanoemulsion), which achieved a C_max of ~ 5.57 ng/mL and AUC of ~ 9.12 h·ng/mL with a tmax of 0.7. These values are remarkably similar to what our 40 mg enhanced liquid produced (C_max 6.2 ng/mL, AUC 20.2 h·ng/mL for 40 mg; if one scales the 30 mg dose in Williams et al. to 40 mg, one would predict ~ 6.2 ng/mL C_max, which is exactly what we observed). This suggests that our enhanced liquid performed on par with the state-of-the-art nanoemulsion tested in Williams et al.’s trial. Meanwhile, our enhanced capsule achieved even higher exposure: C_max 14.1 ng/mL, AUC 38.0 h·ng/mL (albeit with a slightly later tmax of ~ 2 h). Williams et al. did not include a formulation directly comparable to our capsule; their closest analog might be a “CBD powder in water” or a 5% CBD powder, which gave C_max values of only ~ 1.3–2.9 ng/ml far below our capsule’s performance. In fact, our 40 mg capsule outperformed all five formulations tested by Williams et al. in terms of C_max and AUC (which ranged ~ 2.2–5.6 ng/mL and ~ 4.6–9.1 h·ng/mL, respectively, for 30 mg doses. Even accounting for the slightly higher dose in our study (40 vs. 30 mg), the Supra capsule delivered a higher concentration per mg of CBD. It should be noted that Williams et al. administered their preparations after an overnight fast (with no food until 4 h post-dose). In our study, dosing was with a low-fat meal. Typically, fasting yields lower CBD absorption than even a low-fat fed state because some bile release with food can aid in absorption. Thus, differences in conditions should be considered. Nonetheless, this comparison underscores that the CBDNEXT Supra capsule is among the most effective oral CBD formulations documented to date for systemic delivery of CBD.

Another study by Atsmon et al. (2018) examined PTL101, a gelatin matrix pellet formulation of CBD given with food. They reported a C_max of ~ 3.22 ng/mL for a 10 mg dose. If linearly scaled, a 40 mg dose of that formulation might reach a C_max of ~ 12.9 ng/mL, which is close to what we saw for our 40 mg capsule (14.1 ng/mL) under low-fat fed condition. Atsmon’s study used a standard meal (which likely increased absorption relative to fasting). (Atsmon et al. 2018). Taylor et al. reported that shifting from fasted to high-fat fed conditions increased CBD AUC approximately four-fold; the increment from a low-fat to a high-fat meal is smaller (≈ 1.5–2-fold) and was not assessed here, so any extrapolation beyond our data is speculative (Taylor et al. 2018). Thus, had our subjects taken the Supra capsule with a standard or high-fat meal, one might predict even greater exposure (potentially C_max in the tens of ng/mL and AUC approaching those seen with much larger CBD doses under fed conditions. We specifically avoided a high-fat meal in order to evaluate the formulations’ inherent performance and to simulate a likely real-world usage scenario (not every dose of CBD will be taken with a fatty meal in practice). The fact that the Supra formulations still achieved high plasma levels under low-fat conditions is a testament to their effectiveness, and suggests they can reduce the dependence on dietary fat. This could translate to more consistent dosing in patients irrespective of meal content, a significant practical benefit.

It is also informative to compare the performance of our reference product to literature expectations. For a 40 mg plain CBD capsule in a fed (low-fat) state, our reference C_max of ~ 2.4 ng/mL is low but not surprising. Britch et al. (2021) noted that oral CBD exhibits roughly dose-proportional C_max in the 5–120 mg range but requires a high-fat meal to reach its full absorption potential. In fasted or low-fat conditions, peak concentrations around 2–3 ng/mL at a 40 mg dose seem plausible, which is exactly what we observed in some subjects with the reference. The very high variability we observed with the reference (some subjects absorbing almost no CBD) is also in line with known challenges; possibly those individuals had less bile secretion or faster GI transit, etc. Our reference product essentially serves as a worst-case baseline. In real-world use, patients might use oil-based CBD tinctures or gel capsules containing CBD dissolved in oils, which would likely perform somewhat better than our dry powder reference (because oil vehicles can improve absorption modestly). However, even compared to typical CBD oil in medium-chain triglyceride (MCT)– which still has low absolute bioavailability– our Supra capsule would likely show clear superiority.

### Clinical implications

Achieving higher CBD plasma levels can be beneficial for multiple reasons. For instance, therapeutic CBD doses in certain severe conditions (like refractory epilepsy) are on the order of hundreds of milligrams per day, resulting in plasma trough levels of 100–300 ng/mL. Our study dose (40 mg) is much lower, targeting perhaps milder indications (e.g., anxiolytic or analgesic uses) where lower plasma levels (single-digit ng/mL) may suffice or where one would dose repeatedly. The enhanced capsule providing about 5.6× the exposure of regular CBD means patients could potentially take one-third the dose to achieve the same systemic effect. This could significantly reduce treatment costs and improve safety margins, since high doses of CBD are associated with more side effects (e.g., somnolence, diarrhea, and elevated liver enzymes in some cases. In our data, a 40 mg Supra capsule yielded an C max similar to what a ~ 224 mg dose of standard CBD (the reference product) would produce, indicating a meaningful increase in “exposure per milligram” (i.e., potency in terms of delivered dose). This also suggests that for indications requiring higher systemic exposure, the Supra products could reach those levels with fewer pills. Of course, actual therapeutic efficacy needs to be demonstrated in clinical trials; at present, pharmacokinetic enhancement only demonstrates the potential of the novel formulations. We did not measure any direct clinical outcomes, so whether the improved PK profiles translate into superior symptom control or faster onset of relief remains to be determined. Future studies should evaluate whether these pharmacokinetic advantages result in improved clinical outcomes in target patient populations.

### Cortisol and pharmacodynamics

We included cortisol measurements as a probe for any acute physiological effect (i.e. modulation of acute stress responses and cortisol levels) of CBD at these doses. Prior research has shown that CBD can modulate stress responses; for example, Appiah-Kusi et al. (2020) found that a short course of CBD reduced the cortisol increase during a laboratory stress task in individuals at high risk of psychosis. In our healthy volunteers at rest, we did not see any difference in cortisol decline among treatments– all followed the normal circadian pattern of decreasing from morning to afternoon. This indicates that 40 mg CBD, even with enhanced absorption, does not blunt or augment normal cortisol rhythms in the absence of a stressor. However, it is critical to distinguish between cortisol response to pharmacological challenge in the absence of a stressor (as in the current study) and cortisol response to an acute psychosocial stressor. In Appiah-Kusi et al. (2020), all participants were subjected to the Trier Social Stress Test (TSST), and cortisol was measured as a response to that standardized stressor, with and without CBD administration. In our study, cortisol assessment was performed without an exogenous stressor, making the interpretation of cortisol dynamics less clear from a pharmacodynamic standpoint. Accordingly, the current design may be more informative from a safety/tolerability perspective—namely, the absence of hypothalamic-pituitary-adrenal (HPA) axis suppression in healthy participants. Our study was not powered to draw confirmatory conclusions regarding this exploratory cortisol outcome. Hence, while no HPA-axis suppression is reassuring from a safety perspective, further claims about CBD’s modulation of stress or anxiety-related neuroendocrine responses (i.e., CBD might help when cortisol is pathologically elevated or during acute stress, but not otherwise) should be considered within these limitations.

Our finding aligns with the notion that CBD is generally inert in non-challenged physiological states, consistent with its good tolerability and low side-effect profile. It’s worth noting that at much higher doses, CBD has been associated with sedation and possibly slightly reduced cortisol (through indirect CNS effects), but 40 mg is relatively low. Therefore, the lack of a cortisol effect in our study was expected and confirms that any pharmacodynamic impact of our formulations would likely manifest only in appropriate settings (e.g., reduction of pathological symptoms, not in baseline hormone levels of healthy subjects).

### Safety considerations

No new safety signals emerged in this study for the Supra formulations. The types and frequency of AEs were similar to those seen with the reference product. CBD is known to have a favorable safety profile in humans, with only mild to moderate AEs (like tiredness, diarrhea, changes in appetite) typically observed, usually at much higher doses. (Iffland and Grotenhermen 2017) Our observed incidence of mild headache (which may or may not have been directly related to CBD) is consistent with CBD’s benign nature. Importantly, we saw **no** liver enzyme elevations in any subject. In some trials of very high-dose CBD (for example, in epilepsy patients taking ~ 20 mg/kg/day, i.e. gram-level doses), reversible elevations in ALT/AST have been noted, especially when CBD is combined with other medications. At a 40 mg single dose, liver stress was not a concern– and our enhanced formulations did not show any idiosyncratic hepatic signals. One might wonder whether higher bioavailability could impose a greater metabolic burden on the liver, potentially leading to enzyme elevations. Our data suggest that at these modest doses, the liver easily handled the increased load (peak CBD ~ 14 ng/mL, which is still low in absolute terms). All other safety parameters (hematology, renal function, vital signs, ECG) remained normal. There was no evidence of any psychoactive effects or abuse potential– consistent with CBD’s known profile and the lack of THC. In fact, prior studies specifically examining CBD’s abuse liability found no intoxication or reinforcement even at much higher plasma level. The absence of any CNS depression or stimulation in our subjects corroborates that the enhanced formulations, while improving absorption, do not introduce any unexpected effects; they simply deliver CBD more efficiently.

One could argue that a higher C_max might, in theory, cause more transient side effects if such effects are dose-rate related (for example, some individuals report light sedation or dizziness at peak CBD concentrations). We did not systematically observe sedation in our study, though one or two subjects did choose to rest quietly post-dose (but similar behavior occurred even with the reference in some cases). There was no pattern suggesting that the high C_max from the capsule led to more side effects than the reference– if anything, those who absorbed very little CBD with the reference had no pharmacological effect at all (and indeed reported nothing), whereas on the enhanced capsule they achieved therapeutic levels yet still did not report adverse effects, implying that even a ~ 5.7 -fold jump in C_max remained well tolerated. This reinforces the wide therapeutic index of CBD, and suggests that the safety profile of CBD is unlikely to be compromised by enhancing its bioavailability at these levels.

### Study limitations

This study has some limitations to consider. First, the sample size was relatively small (*N* = 9 completers for PK analysis), which is acceptable for a pilot bioavailability study but limits the ability to detect rare AEs or to generalize variability estimates to broader populations. However, the crossover design mitigates inter-subject differences since each subject served as his own control, strengthening the comparison despite the small sample. Second, all participants were male, and it is known that sex can influence cannabinoid pharmacokinetics. Some data suggest females might achieve higher CBD plasma levels than males due to differences in body fat distribution or hormone level. We chose to restrict the enrollment to males in this initial study to avoid potential sex-related variability in this small sample. Now that the safety and bioavailability outcomes are favorable, future studies should include female participants to confirm that the formulations perform equally well in women (they likely will, and perhaps even with slightly higher absorption as noted in some reports where women had ~ twofold higher CBD exposure than men under certain conditions). (Stott et al. 2013) Third, we did not include a fasting arm or a high-fat meal arm for direct comparison. Our focus was on the low-fat fed state, which is somewhat uncommon in regulatory bioavailability studies (they often examine fasting or high-fat extremes). We justified this design as a way to simulate normal breakfast conditions and to test the formulations without the maximal food effect. It would be informative in the future to evaluate the Supra capsule and liquid under strict fasting and high-fat meal conditions as well. It’s possible that under fasting conditions, the reference would perform even worse (perhaps C_max < 2 ng/mL), whereas the Supra formulations might also decrease somewhat but would still be significantly higher than the reference– that would demonstrate the extent to which the formulation itself drives absorption versus reliance on bile/fat. Conversely, with a high-fat meal, all formulations would likely show increased absorption. Gathering such data would help delineate how robust the formulation advantage is across nutritional states. Fourth, we only evaluated single-dose pharmacokinetics. We do not know if there would be any accumulation or induction/inhibition effects on repeated dosing. CBD can induce certain CYP enzymes at high doses or interact with other drugs (for example, it can inhibit CYP2C19 and CYP3A4 in vitro), but at 40 mg/day such interactions are minimal. Still, chronic dosing studies with these formulations will be needed to ensure no unexpected issues (e.g., whether the surfactant might affect absorption of co-administered medications– although the quantities per dose are small). (Bansal et al. 2020)

### Performance of capsule vs. liquid

It is interesting that the enhanced capsule outperformed the enhanced liquid in terms of total exposure. One might intuitively expect a liquid nanoemulsion to be absorbed as completely as, or faster than, a capsule that has to dissolve, but our data showed the capsule yielding higher C_max and AUC. One hypothesis is that the capsule’s contents, once released in the stomach, form a slightly different emulsion that perhaps persists longer through the intestines due to the presence of the solid matrix (silica/dextrin)– effectively providing a sustained solubilization as the dosage form travels through the gut. The liquid, being pre-dispersed, might empty from the stomach and be absorbed very quickly, but any CBD not absorbed in the upper GI could precipitate later or might not have the “carrier” matrix to keep it solubilized further down the intestine. In other words, the capsule’s powder formulation may interact with gastric fluids more gradually, continually releasing solubilized CBD along the GI tract, whereas the liquid delivers a bolus of solubilized CBD upfront. It’s also conceivable that the liquid’s very rapid absorption led to a higher first-pass extraction: if a large fraction of the dose is absorbed all at once in the proximal small intestine, the liver may extract more of it on first pass, whereas a slower, more prolonged input (as possibly from the capsule) might allow a greater portion of CBD to escape first-pass metabolism via distribution before hepatic clearance. This is speculative, but in line with the idea that extremely rapid absorption could paradoxically result in more metabolism. Regardless, the differences between capsule and liquid were not enormous– the capsule’s AUC was roughly 1.9× the liquid’s, and C_max ~ 2.3×. These differences were noticeable with our sensitive analytical method, but in practical terms both test formulations delivered vastly more CBD than the reference. From a product development perspective, this raises the question: if only one formulation were to be advanced, the capsule might be preferable due to its higher bioavailability and greater convenience (solid dose stability, ease of handling, etc.). On the other hand, the liquid’s advantage is the faster onset. In some indications (e.g., acute anxiety episodes or breakthrough pain), a faster onset could be valuable. Potentially, both formats could be utilized in different scenarios– the liquid as a “rescue” or rapid-relief option, and the capsule as a routine maintenance dose for sustained coverage based on further studies. The encouraging part is that both were well tolerated.

### Potential dose reduction and cost savings

Given the improved bioavailability observed, patients may achieve the same systemic exposure with a lower dose when using these new formulations. This is economically significant since high-purity CBD is expensive. For example, if a patient typically needs 100 mg of standard CBD oil to attain a certain therapeutic level, they might only need on the order of ~ 20 mg of the Supra capsule to reach a similar plasma concentration. That’s roughly a 80% reduction in dose (and presumably in cost) for each administration. Over time, this could make CBD therapy more accessible and affordable. Additionally, lower doses mean less ingestion of carrier oils or excipients, potentially reducing caloric intake or gastrointestinal side effects associated with those vehicles (for instance, some individuals experience diarrhea from the large amounts of MCT oil used in high-dose CBD tinctures. (Fasinu et al. 2016) By delivering more drug with less excipient, the new formulations can improve the overall treatment experience.

### Future directions

The promising results of this study pave the way for further research and development. Next steps could include a repeated-dose study to see if steady-state is achieved by approximately five half-lives (~ 5–7 days of daily dosing) and whether accumulation is linear (likely yes, given CBD’s kinetics are linear at this dose). Additionally, a clinical trial in a relevant patient population (e.g., an anxiety disorder or chronic pain condition) comparing equal doses of the Supra capsule vs. a traditional CBD product would be valuable to confirm if the improved PK translates into better symptom control or faster onset of relief. Studying the effect of a high-fat meal on the Supra formulations would also be informative: does a high-fat meal boost their absorption even further, or are they already so efficient that food adds little? If the latter, it means these formulations can free patients from having to coordinate dosing with meals– an advantage in convenience and adherence. From a regulatory standpoint, if one were to develop the Supra capsule as a pharmaceutical product, one strategy might be to show that a lower dose of it is bioequivalent to a higher dose of an approved CBD product (for example, demonstrating that a 50 mg Supra capsule is ≈ bioequivalent to 100 mg of Epidiolex^®^ under certain conditions). That could support labeling that allows lower dosing for the same effect. Our study provides initial dose-exposure ratios that could inform such bridging strategies.

## Conclusions

In summary, this single-dose crossover study demonstrated that two novel bioavailability-enhanced CBD formulations (Supra capsule and Supra liquid) markedly improve the pharmacokinetic profile of orally administered CBD in healthy volunteers under low-fat fed conditions. Both formulations achieved significantly higher plasma CBD concentrations, faster absorption, and more consistent exposure compared to the reference product (a standard high-purity CBD isolate capsule). The 40 mg Supra capsule yielded the greatest C_max and AUC, approximately 5–6 times and 3–4 times that of the reference, respectively, and also showed the lowest variability. The 40 mg Supra liquid provided the most rapid absorption (tmax ~ 1 h) and about 2.5-fold higher C_max and 1.7-fold higher AUC than the reference. All formulations were well tolerated, with no serious adverse effects and no clinically significant changes in safety parameters. The enhanced absorption did not cause any abnormal acute pharmacodynamic effects (as evidenced by normal cortisol profiles and clinical observations). These findings indicate that the proprietary formulation technology (combining CBD with specific surfactants and acids) is highly effective in overcoming CBD’s oral bioavailability limitations.

From a practical perspective, both the solid and liquid new formulations offer substantial advantages: the capsule form appears ideal for maximizing overall exposure (and potentially providing longer-lasting plasma levels), while the liquid ensures a very quick onset of absorption. The ability to attain high CBD levels without a high-fat meal means more flexible dosing in real-world use and potentially reduced variability due to fed/fasted differences. Given the favorable safety profile observed, these formulations could allow patients to use lower doses of CBD to achieve the same therapeutic exposure, enhancing cost-effectiveness and possibly adherence.

The solid CBDNEXT Supra capsule is particularly promising as a convenient, stable oral dosage form that delivered the highest bioavailability in this study. It may represent a more “patient-friendly” and efficient CBD product for clinical use, meriting further development and clinical testing. Future clinical trials should evaluate the efficacy of these enhanced formulations in specific conditions (e.g., anxiety, epilepsy, pain) to determine if the pharmacokinetic advantages translate into improved therapeutic outcomes.

In conclusion, the novel bioavailability-enhanced CBD oral formulations significantly outperformed the reference product (a standard CBD isolate capsule) in systemic exposure under fed conditions. The results support their potential to optimize CBD therapy by providing faster and greater delivery of the active compound. This study contributes to the growing efforts to optimize cannabinoid delivery and paves the way for more efficient and reliable oral CBD treatments. With robust pharmacokinetic advantages and maintained safety, the CBDNEXT Supra capsule (and liquid) can be considered next-generation oral CBD products that address CBD’s bioavailability limitations and hold promise for broad clinical use.

## Electronic supplementary material

Below is the link to the electronic supplementary material.

Supplementary Material 1

## Data Availability

The data supporting the findings of this study are available within the article, further inquiries can be directed to the corresponding author.
