# Supplementary material for: Randomized single-dose crossover comparative bioavailability study of two novel oral cannabidiol (CBD) formulations in healthy volunteers under fed conditions, compared to a standard CBD isolate capsule
Source: J Cannabis Res. 2025 Aug 6;7:54. doi: 10.1186/s42238-025-00312-9 (PMC12330118; doi:10.1186/s42238-025-00312-9)
Supplement: Supplementary file 1 — Supplementary Material 1 [file 42238_2025_312_MOESM1_ESM.docx]

**Supplementary Table S1.** Individual vital-sign measurements (systolic/diastolic blood pressure in mmHg, heart rate in bpm, respiratory rate) for all 12 dosed subjects at key time-points in each treatment period

|  |  | **Vital signs: SBP/DBP (mmHg) ‖Heart rate (bpm)‖ Respiratory rate** | | | | | | | | |
| --- | --- | --- | --- | --- | --- | --- | --- | --- | --- | --- |
| **Subj ID** | **Treatment**  **<sup>a</sup>** | **Screening** | **Pre-dose** | **1 h post** | **2 h post** | **4h post** | **6h post** | **12h post** | **23h post** | **72h post** |
| 01 | Test1 (capsule)  Test2 (Liquid)  Test3 (capsule) | 130/87 ‖96‖ 17 | 121/72 ‖71‖ 15  (NA)  (NA) | 107/71 ‖76‖ 14  (NA)  (NA) | 105/64 ‖74‖ 14  (NA)  (NA) | 110/70 ‖72‖ 16  (NA)  (NA) | 120/80 ‖ 81‖ 14  (NA)  (NA) | 110/70 ‖ 74‖ 16  (NA)  (NA) | 117/75 ‖ 76‖ 15  (NA)  (NA) | 110/70 ‖ 77‖ 16  (NA)  (NA) |
| 02 | Test1 (capsule)  Test2 (Liquid)  Test3 (capsule) | 118/79 ‖80‖ 14 | 108/68 ‖68‖ 14  115/70 ‖62‖ 13  121/70 ‖70‖ 13 | 110/70 ‖77‖ 16  130/72 ‖74‖ 18  120/70 ‖71‖ 14 | 113/72 ‖82‖ 18  130/80 ‖79‖ 18  116/68 ‖70‖ 14 | 114/72 ‖66‖ 14  108/78 ‖60‖ 16  117/75 ‖79‖ 14 | 112/72 ‖76‖ 16  124/71 ‖79‖ 18  129/77 ‖78‖ 14 | 113/71 ‖81‖ 14  115/79 ‖75‖ 16  113/71 ‖75‖ 16 | 115/79 ‖80‖ 17  122/81 ‖76‖ 14  116/80 ‖76‖ 16 | 119/70 ‖61‖ 15  120/70 ‖77‖ 15  113/70 ‖69‖ 15 |
| 03 | Test1 (capsule)  Test2 (Liquid)  Test3 (capsule) | 125/81 ‖78‖ 14 | (NA)  118/71 ‖70‖ 15  118/71 ‖80‖ 17 | (NA)  125/80 ‖80‖ 14  117/70 ‖77‖16 | (NA)  124/81 ‖86‖ 18  113/69 ‖80‖ 16 | (NA)  117/76 ‖80‖ 14  124/75 ‖80‖ 18 | (NA)  130/80 ‖84‖ 16  128/80 ‖74‖ 17 | (NA)  127/82 ‖75‖ 18  121/82 ‖80‖ 17 | (NA)  111/70 ‖75‖ 15  118/76 ‖72‖ 15 | (NA)  122/80 ‖70‖ 12  114/74 ‖71‖ 18 |
| 04 | Test1 (capsule)  Test2 (Liquid)  Test3 (capsule) | 137/87 ‖ 79‖ 15 | 121/85 ‖9‖ 15  115/72 ‖89‖ 14  109/69 ‖79‖ 15 | 115/68 ‖75‖ 17  110/74 ‖75‖ 13  130/82 ‖76‖ 18 | 111/77 ‖65‖ 18  115/81 ‖78‖ 13  131/84 ‖85‖ 16 | 100/60 ‖62‖ 17  119/80 ‖80‖ 13  130/80 ‖77‖ 18 | 130/80 ‖ 80‖ 19  124/80 ‖ 77‖ 13  130/84 ‖ 76‖ 18 | 113/70 ‖ 72‖ 15  122/84 ‖ 82‖ 14  116/84 ‖ 88‖ 16 | 114/71 ‖ 78‖ 15  113/71 ‖ 71‖ 12  122/79 ‖ 77‖ 15 | 120/70 ‖ 71‖ 15  112/70 ‖ 80‖ 18  114/74 ‖ 73‖ 14 |
| 05 | Test1 (capsule)  Test2 (Liquid)  Test3 (capsule) | 118/76 ‖73‖ 15 | 113/73 ‖63‖ 14  110/72 ‖65‖ 14  120 /77 ‖80‖ 15 | 110/70 ‖72‖ 18  105/71 ‖72‖ 19  110/72 ‖85‖ 12 | 105/64 ‖71‖ 16  102/75 ‖67‖ 19  115/78 ‖84‖ 14 | 107/70 ‖88‖ 18  100/62 ‖60‖ 18  112/81 ‖82‖ 14 | 116/82 ‖84‖ 14  106/79 ‖71‖ 18  115/78 ‖82‖ 14 | 102/66 ‖76‖ 14  110/67 ‖69‖ 13  120/80 ‖76‖ 17 | 120/80 ‖79‖ 17  124/79 ‖80‖ 16  120/80 ‖82‖ 16 | 118/70 ‖62‖ 13  110/70 ‖77‖ 13  110/71 ‖83‖ 17 |
| 06 | Test1 (capsule)  Test2 (Liquid)  Test3 (capsule) | 115 72 ‖78‖ 16 | 114/70 ‖80‖ 12  120/73 ‖84‖ 13  126 / 81 ‖69‖ 16 | 124 /77 ‖79‖ 18  116 /80 ‖83‖ 14  106/72 ‖75‖ 14 | 116/72 ‖77‖ 18  122/83 ‖79‖ 13  130/82 ‖82‖ 14 | 130/75 ‖76‖ 19  120/83 ‖81‖ 15  125/84 ‖86‖ 16 | 125 / 80 ‖79‖ 17  122 / 81 ‖ 85‖ 15  128 / 80 ‖76‖ 20 | 115 / 65 ‖67‖ 13  124 / 85 ‖87‖ 18  120 / 80 ‖87‖ 20 | 115 / 68 ‖70‖ 15  118 / 76 ‖73‖ 20  113 / 76 ‖82‖ 16 | 112/72 ‖74‖ 14  119/70 ‖65‖ 14  110 / 70 ‖77‖ 13 |
| 07 | Test1 (capsule)  Test2 (Liquid)  Test3 (capsule) | 113/73 ‖79‖16 | (NA)  117 / 76 ‖70‖ 14  (NA) | (NA)  108/64 ‖67‖ 16  (NA) | (NA)  102/64 ‖66‖ 18  (NA) | (NA)  118/80 ‖86‖ 14  (NA) | (NA)  100 / 65 ‖67‖ 18  (NA) | (NA)  117 / 72 ‖85‖ 18  (NA) | (NA)  115 / 70 ‖73‖ 17  (NA) | (NA)  115 / 70 ‖ 62‖ 18  (NA) |
| 08 | Test1 (capsule)  Test2 (Liquid)  Test3 (capsule) | 113/74 ‖73‖ 15 | 113 / 70 ‖79‖ 15  105 / 65 ‖64‖ 15  122 / 82 ‖71‖ 15 | 117 /73 ‖70‖ 15  110 /70 ‖62‖ 14  100/60 ‖72‖ 18 | 118/73 ‖71‖ 12  112/78 ‖75‖ 14  113/73 ‖69‖ 17 | 113/78 ‖80‖ 14  110/66 ‖64‖ 20  100/61 ‖67‖ 16 | 109 / 68 ‖73‖ 13  110 / 71 ‖88‖ 16  100 / 60 ‖67‖ 18 | 117 / 73 ‖76‖ 14  109 / 68 ‖77‖ 16  120 / 71 ‖70‖ 17 | 110 / 70 ‖77‖ 18  115 / 82 ‖81‖ 15  113 / 69 ‖66‖ 14 | 122 / 80 ‖ 75‖ 16  120 / 70 ‖ 70‖ 16  112 / 72 ‖ 74‖ 17 |
| 09 | Test1 (capsule)  Test2 (Liquid)  Test3 (capsule) | 117/71 ‖73‖ 14 | 110 / 63 ‖68‖ 15  125 / 79 ‖83‖ 12  120 / 72 ‖89‖ 14 | 108/66 ‖83‖ 16  115 /74 ‖71‖ 18  120/82 ‖80‖ 14 | 111/72 ‖68‖ 16  110/65 ‖75‖ 16  119/72 ‖80‖ 14 | 100/62 ‖84‖ 18  110/70 ‖72‖ 17  115/75 ‖83‖ 13 | 114 / 65 ‖83‖ 14  105 / 75 ‖76‖ 16  123 / 77 ‖79‖ 14 | 120 / 81 ‖68‖ 14  121 / 80 ‖79‖ 16  130 / 85 ‖88‖ 18 | 121 / 79 ‖77‖ 18  126 / 77 ‖71‖ 15  118 / 82 ‖86‖ 17 | 114 / 70 ‖62‖ 15  113 / 74 ‖71‖ 16  116 / 70 ‖61‖ 15 |
| 10 | Test1 (capsule)  Test2 (Liquid)  Test3 (capsule) | 120/80 ‖78‖ 17 | 113 / 70 ‖70‖ 13  118 / 76 ‖73‖ 14  116 / 70 ‖75‖ 16 | 112 /70 ‖75‖ 13  105/62 ‖62‖ 16  116/80 ‖65‖ 17 | 115/80 ‖70‖ 13  112/72 ‖77‖ 18  117/70 ‖76‖ 18 | 120/79 ‖80‖ 14  116/80 ‖66‖ 14  106/63 ‖65‖ 16 | 120 / 85 ‖83‖ 14  120 / 80 ‖77‖ 16  130 / 70 ‖72‖ 18 | 118 / 76 ‖74‖ 16  114 / 72 ‖73‖ 18  111 / 64 ‖65‖ 13 | 115 / 75 ‖85‖ 14  120 / 80 ‖83‖ 15  128 / 84 ‖77‖ 15 | 125 / 80 ‖ 71‖ 15  122 / 80 ‖ 85‖ 17  120 / 70 ‖ 71‖ 15 |
| 11 | Test1 (capsule)  Test2 (Liquid)  Test3 (capsule) | 130/70 ‖77‖ 14 | 118 / 82 ‖81‖ 13  120 / 79 ‖80‖ 15  107 / 71 ‖71‖ 14 | 125/79 ‖67‖ 18  126 /80 ‖78‖ 15  110/70 ‖76‖ 18 | 107/78 ‖62‖ 17  123/78 ‖83‖ 14  130/82 ‖80‖ 16 | 101/68 ‖60‖ 17  116/74 ‖78‖ 13  112/74 ‖76‖ 16 | 108 / 74 ‖67‖ 17  118/ 69 ‖80‖ 13  110 / 70 ‖72‖ 14 | 120 / 80 ‖ 79‖ 17  124 / 84 ‖ 68‖ 14  110 / 65 ‖ 78‖ 16 | 110 / 68 ‖66‖ 14  120 / 80 ‖79‖ 20  127 / 83 ‖85‖ 14 | 110 / 70 ‖71‖ 17  113 / 70 ‖65‖ 14  118 / 70 ‖62‖ 15 |
| 12 | Test1 (capsule)  Test2 (Liquid)  Test3 (capsule) | 139/86 ‖89‖ 15 | 112 / 70 ‖67‖ 18  110 / 73 ‖79‖ 14  103 / 64 ‖62‖ 15 | 118/80 ‖79‖ 18  119 /73 ‖80‖ 15  116/80 ‖88‖ 18 | 125/72 ‖80‖ 18  116/75 ‖85‖ 13  113/67 ‖86‖ 14 | 120/60 ‖80‖ 16  121/76 ‖85‖ 14  113/62 ‖82‖ 18 | 124 / 73 ‖80‖ 19  120 / 77 ‖85‖ 14  112 / 74 ‖84‖ 20 | 123 / 85 ‖82‖ 16  128 / 86 ‖87‖ 18  116 / 80 ‖75‖ 20 | 130 / 81 ‖82‖ 15  125 / 84 ‖88‖ 18  121 / 77 ‖71‖ 16 | 120 / 70 ‖77‖ 14  126 / 88 ‖87‖ 12  112 / 72 ‖79‖ 18 |
